# Bibliographical Department

**Published:** 1880-12

**Authors:** 


### BIBLIOGRAPHICAL DEPARTMENT.

"Nothing extenuate, nor set down aught in malice."

The Art of Prolonging Life.—By Christopher William Heufeland. Edited by Erasmus Wilson, M. D., author of a system of Human Anatomy, etc., etc., from the last London edition, published by Lindsay & Blakiston, Philadelphia, 1880.

The above is a charmingly written little volume of 298 pages, and should be read by all who would live long and well. It is gotten up in the publisher's usual neat and attractive style, and can be had in this city at Messrs. Cushing & Bailey's, Baltimore st.

Another one of the *American Health Primers* has been sent to us by the publisher, Presley Blackiston, Esq., Philadelphia. This one is on *School and Industrial Hygiene*. By D. F. Lincoln, M. D., Chairman of Health Social Science Association. This little brochure is cheerfully recommended to the careful perusal of parents and *teachers* of schools for children.

We have received from Prof. Joseph T. Johnson, M. D., the following re-prints, viz: *A Case of Head and Foot Presentations.* Fracture of the spine in Utero. *On the treatment of Placenta Prævia: Mismanaged Labor the source* of much of the gynecological practice of the present day; and an Introductory Address before the Washington Training School for Nurses. All instructive and well written pamphlets.

Surgical Treatment of Naso-Pharangeal Cartarrh. By D. H. Goodwillie, M. D., D. D. S., of New York City, has also been received. A valuable pamphlet.

Explanation of a Simple Method for the Diagnosis for Organic Valvular Diseases of the Heart.—By F. Peyre Porcher, M. D., Professor of Materia Medica and Therapeutics in the Medical College of South Carolina, Associate Fellow of the College of Physicians of Philadelphia.

The foregoing is an extract from the American Journal of the Medical Sciences, and, like everything emanating from the pen of its accomplished and talented author, is worthy the perusal of any one who desires to find in a small compass all that is really important in reaching a correct diagnosis in the trouble to which it relates.

The Journal of Nervous and Mental Diseases edited by J. S. Jewell, M. D., Professor of Nervous and Mental diseases, in Chicago Medical College, and H. M. Bannister, M. D.; Associate editors, W. A. Hammond, M. D., Meredith Clymer, M. D., New York,

S. Weir Mitchell, M. D., Philadelphia. October, 1880, Office of publication, Chicago, 70 Munroe st. Branch office, New York, G. P. Putnam's Sons, 182 Fifth Avenue.

Subscription, $5 ; Single Numbers, $1.50

It is hardly necessary to say, after giving the names of the editorial corps, that this is one of the ablest edited Journals in the English language, on this side the water at least. As such it should have a large circulation everywhere. We cheerfully give it a place on our list of exchanges.

Prof. William Goodell, M. D., sends us an extract from the Medical Society of the state of Pensylvania on the *Radical Treatment of Fibroid Tumors of the Womb*, which we have read with much interest.

A Practical Treatise on Mechanical Dentistry. By Joseph Richardson, D. D. S., M. D., Professor of the Principles of Prosthetic Dentistry, in the Indiana Dental College; formerly Professor of Mechanical Dentistry and Metallurgy in the Ohio College of Dental Surgery. Third edition, revised and enlarged, with one hundred and eighty-five illustrations. Philadelphia: Lindsay and Blackiston, 1880. The rapidity with which this work has passed from one edition to another speaks more in its behalf than anything we could say in commendation of it. That it will prove a valuable addition to the library of the Dentist who desires to understand the mechanical department of his calling we feel fully assured, as its matter and illustrations are both of the first order. The publishers h ve also performed their part in their usual excellent style.

Price in cloth, $4.00; Leather, $4.75. For sale in Baltimore by Cushings and Bailey.

We are in the receipt of the Records of the Connecticut Valley Dental Society from October, 1876, to January, 1879, inclusive, with list of officers and members, Springfield, Mass., 1880. We find much to interest dentists in this little volume.

We have been favored also with the Transactions of the Illinois State Dental Society, 16th Annual Session held at Bloomington, Ill., May 11, 1880, which is another collection of valuable facts that cannot fail to interest the reader.

The following interesting reports were received just as this issue was going to the press, viz : "*A Case of Combined Intra-uterine and Abdominal Twin Pregnancy.*" The first child naturally at eight months, and the second delivered at ten by laparotomy. By H. P. C. Wilson, M. D., etc. "*A Treatise on Hip Joint Disease.*" By J. D. Roberts, M. D.

